# Down-regulation of miR-29b in carcinoma associated fibroblasts promotes cell growth and metastasis of breast cancer

**DOI:** 10.18632/oncotarget.17136

**Published:** 2017-04-16

**Authors:** Yonglei Liu, Jingling Zhang, Xiangjun Sun, Quanping Su, Cuiping You

**Affiliations:** ^1^ Research Center, Linyi People's Hospital, Shandong, China; ^2^ Zhongshan Hospital, Fudan University, Shanghai, China; ^3^ Department of Surgery, Linyi People's Hospital, Shandong, China

**Keywords:** breast cancer, miR-29b, fibroblasts, CCL11, CXCL14

## Abstract

Carcinoma associated fibroblasts (CAFs) play important roles in breast cancer development and progression. Recent studies show that microRNAs (miRNAs) are the main regulators in CAFs. MiR-29b is one of the significant down-regulated miRNAs in CAFs from the miRNA screening. The role of miR-29b in the interaction between CAFs and breast cancer is still unclear. In the present study, we investigated the effects of CAFs on breast cancer cell proliferation and metastasis regulated by miR-29b. We found that fibroblasts activated by co-cultured breast cancer cells produced higher levels of some chemokines like CCL11, CXCL14, which accelerated breast cancer cell growth and induced drug resistance and metastasis. Increased miR-29b expression in activated fibroblasts could suppress the activating p38-STAT1 signal pathway in breast cancer cells. We also found that the expression of CCL11 and CXCL14 could be regulated by miR-29b in CAFs. Our results illustrate that down-regulation of miR-29b in CAFs plays an important role in tumor stroma by activating p38-STAT1 in breast cancer cells. The study indicates that cancer cells and fibroblasts interaction promotes breast cancer cell growth, drug resistance, migration and invasion due to the lack of miR-29b expression in CAFs.

## INTRODUCTION

The tumor microenvironment plays very important roles in the development and progression of cancer. Cancer tissues consist of various cells include cancer cells, fibroblasts, endothelial cells, pericytes, immune cells and various bone marrow-derived progenitor cells [1–2]. Fibroblasts are the most enriched cells in tumor stroma. The activated cancer-associated fibroblasts (CAFs) in the cancer niche build a permissive and supportive microenvironment for tumor development. Fibroblasts play important roles in cancer progression including metabolism, metastasis, proliferation, anti-apoptosis, angiogenesis and chemo-resistance by interaction with cancer cells or other cells [3–5]. Fibroblasts could affect behaviors of cancer cells by activating signal pathways, microRNAs (miRNAs), and soluble molecules and so on.

MiRNAs are a type of non-coding small RNA molecules that negatively regulate gene expression at a posttranscriptional level by binding to the 3′ UTR of its target genes [6]. More and more research focus on the roles of miRNAs in fibroblasts [7–9]. Fibroblasts secrete a variety of chemokines, growth factors, cytokines, which can be regulated by miRNAs [6–9]. Down-regulation of miR-149, miR-21, miR-409, miR-26b, miR-205, miR-31 and up-regulation of miR-34b and miR-34c expression could transform resident fibroblasts to carcinoma associated fibroblasts (CAFs) [10–17]. The reported miRNAs could regulate gene expression in fibroblasts and then control cancer development and progression by regulating cytokines or chemokines. Chemokines are critical signaling mediators, which involve in the communication between the cancer cells and fibroblasts through binding to their receptors [18]. Cell survival, proliferation, stem cell properties, ECM attachment, adhesion, metastasis in tissues or to other organs including proteolysis of the basement membrane, locomotion and colony formation will be changed according to the signals from the CAFs [19].

Our previous screening data showed that miR-29b is one of the significant down-regulated miRNAs in CAFs. Our study is to determine how miR-29b influences chemokine secretion of CAFs and its role in the regulation of proliferation, drug resistance and metastasis of breast cancer cells.

## RESULTS

### MiR-29b expression down-regulates in CAFs

**Figure 1:**
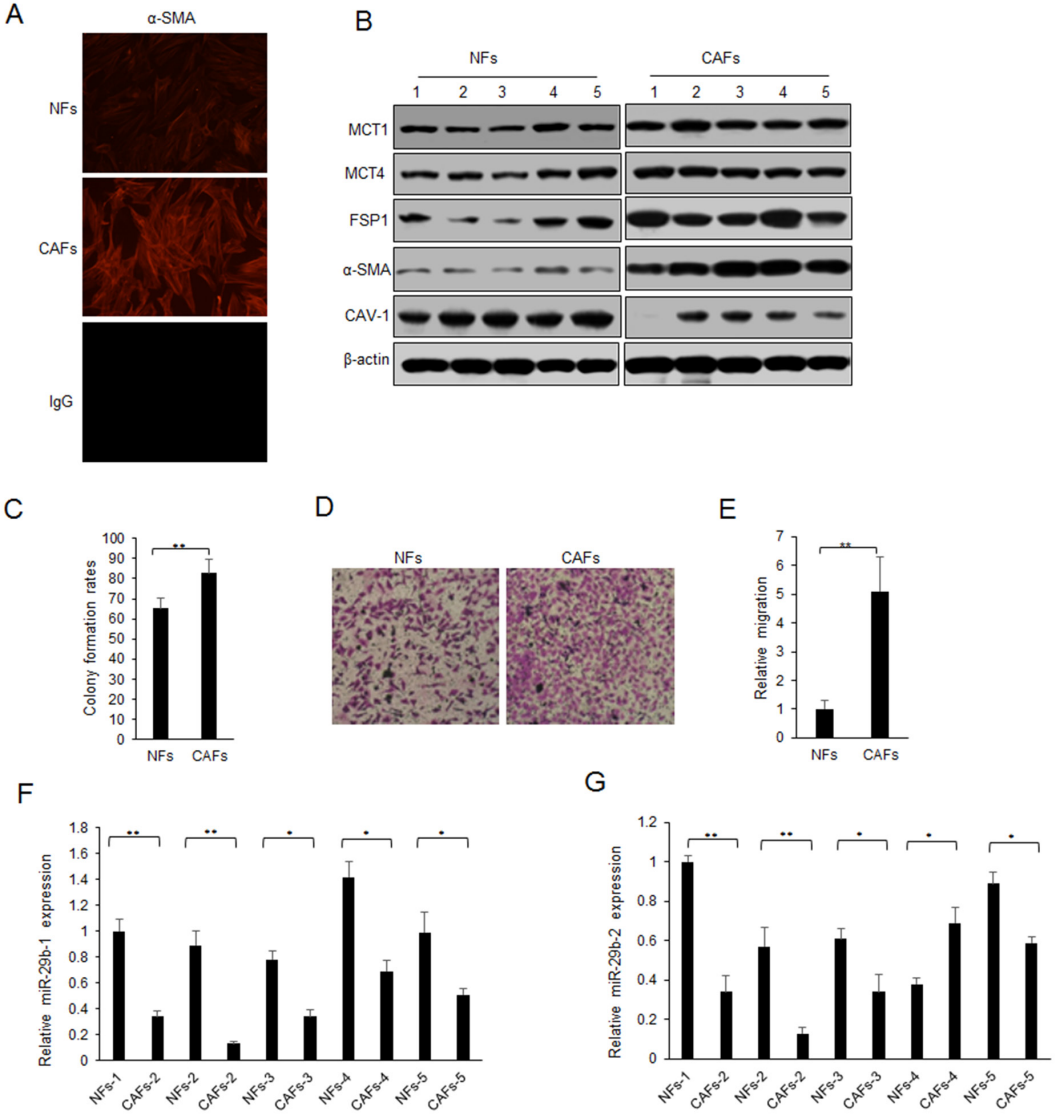
Lack of miR-29b expression in CAFs **(A)** α-SMA expression in CAFs and NFs by immunofluorescence assay. **(B)** Markers of CAFs and NFs from breast cancer tissues were examined by western blotting. **(C)** Colony formation rates of SKBR3 cells with CM treatment from NFs or CAFs. **(D)** Migration ability of SKBR3 cells with CM treatment from NFs or CAFs. **(E)** Data analysis from D. **(F-G)** miR-29b-1-5p and miR-29b-2-5p expression in CAFs and NFs by real time RT-PCR. The data were shown as means±s.d. collected from three independent experiments. *: *p*<0.05, **: *p*<0.01, and -: no significance.

CAFs and normal fibroblasts (NFs) were isolated from the tissues of breast cancer. α-SMA is the most common marker of CAFs, and it was significantly up-regulated in CAFs than in NFs using immunofluorescence assay (Figure 1A). The markers of CAFs including MCT1, MCT4, α-SMA and FSP1 were examined by western blotting. It was found that MCT1, MCT4, α-SMA and FSP1 protein levels were higher than them in NFs, but CAV-1 was significantly down-regulated in CAFs (Figure 1B). Cell proliferation was assayed for SKBR3 with conditioned medium (CM) from NFs or CAFs treatment by colony formation assay (Figure 1C). The results indicated that CAFs were different from NFs, and CAFs showed the features of the activated fibroblasts. It was also shown that CAFs promoted cell migration of SKBR3 cells (Figure 1D–1E).

To know the role of miRNAs in CAFs, miRNA array was performed to find the significant miRNAs. MiR-29b is one of the down-regulated miRNA in CAFs (Supplementary Table 1). It was also found that miR-29b including miR-29b-1-5p (miR-29b-1) and miR-29b-2-5p (miR-29b-2) were down-regulated in CAFs compared to NFs (Figure 1F–1G). These indicated that miR-29b may play a suppressing role in CAFs.

### CAFs with miR-29b inhibits breast cancer cellular growth

**Figure 2:**
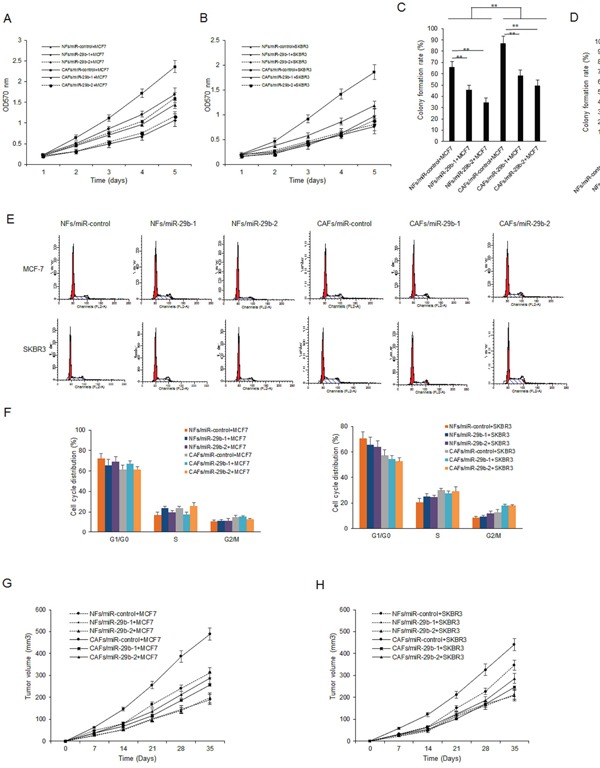
CAFs enhances breast cancer cellular viability by down-regulation of miR-29b **(A-B)** CAFs were transfected with miR-29b-1 or miR-29b-2 mimics for 24h and then co-cultured with breast cancer cells. MTT assay was used to test cell proliferation at day 0, 1, 2, 3, 4 and 5. **(C-D)** CAFs were transfected with miR-29b-1 or miR-29b-2 mimics for 24h and then co-cultured with breast cancer cells. Colony formation assay was used to evaluate cell survival ability. **(E)** Breast cancer cells were transfected with miR-29b-1 or miR-29b-2 mimics and flow cytometry was used to analyze cell cycle distribution of breast cancer cells. **(F)** Ratio of cell cycle distribution from G. **(G-H)** Tumor growth *in vivo* in the model of breast cancer using parent cells (MCF7 and SKBR3), or cancer cells combined with CAFs, or cancer cells combined with CAFs/miR-29-1, or CAFs/miR-29-2. The data were shown as means±s.d. collected from three independent experiments. *: *p*<0.05, **: *p*<0.01.

To further investigate the role of miR-29b in CAFs on breast cancer cells, CAFs cells were transfected with miR-29b-1 or miR-29b-2 and their expression increased (Supplementary Figure 1). Cell proliferation was examined by MTT assay. It was found that miR-29b-1 or miR-29b-2 could inhibit cell survival in three breast cancer cell lines including MCF-7 and SKBR3 (Figure 2A–2B). Next, colony formation assay was used for evaluate cell survival ability. It was shown that miR-29b-1 or miR-29b-2 overexpression in CAFs reduced the number of colonies of breast cancer cells, specially, in MCF-7 cells (Figure 2C–2D). Cell cycle, reflecting the cell proliferation, was analyzed by flow cytometry in breast cancer cells after co-culturing CAFs with miR-29b-1 or miR-29b-2 overexpression. The result showed that miR-29b-1 and miR-29b-2 could decrease the ratio of G1, G2/M phase and increase S phase in MCF-7 and SKBR3 cells (Figure 2E–2F). Breast cancer models were set up using nude mice injected with MCF-7 cells and used to verify the effect of miR-29b-1 or miR-29b-2 on cell growth *in vivo*. It was found that MCF-7 cells mixed with CAFs could accelerate tumor growth than MCF-7 cells. MCF-7 cells mixed with miR-29b-1 or miR-29b-2 overexpressed CAFs attenuated tumor growth (Figure 2G). There was a similar result in breast cancer models using BT474 cells (Figure 2H). These data suggested that CAFs with miR-29b-1 or miR-29b-2 overexpression suppressed breast cancer cell growth *in vitro* and *in vivo*.

### MiR-29b regulates the expression of cytokines in CAFs

**Figure 3:**
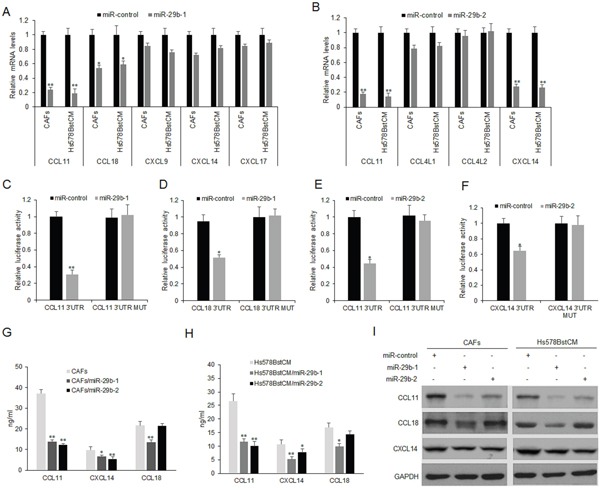
MiR-29b regulates CCL11 and CXCL14 expression in fibroblasts **(A)** miR-29b-1 restoration down-regulated CCL11 and CCL18 mRNA in CAFs and Hs578Bst fibroblasts treated with conditioned medium from cancer (Hs578BstCM). Cells were transfected with miR-29b-1 or miR-control for 48 hours, then collected for real-time PCR. **(B)** miR-29b-2 restoration down-regulated CCL11 and CXCL14 mRNA in CAFs and Hs578BstCM. Cells were transfected with miR-29b-2 or miR-control for 48 hours, then collected for real-time PCR. **(C-D)** miR-29b-1 suppressed the expression of a luciferase reporter gene harboring the 3′-UTR of CCL11 or CCL18. **(E-F)** miR-29b-2 suppressed the expression of a luciferase reporter gene harboring the 3′-UTR of CCL11 and CXCL14. **(G)** Cells were transfected with miR-29b-1, miR-29b-2 or miR-control for 48 hours, then collected for ELISA. **(I)** Cells were transfected with miR-29b-1, miR-29b-2 or miR-control for 48 hours, then collected for western blotting. The data were shown as means±s.d. collected from three independent experiments. *:*p*<0.05, **: *p*<0.01.

The bioinformatics analysis showed that 3′-UTRs of the CCL11, CCL18, CXCL9, CXCL14 and CXCL17 contain the binding sites with miR-29b-1 (Supplementary Figure 2). 3′-UTRs of the CCL11, CCL4L1, CCL4L2 and CXCL14 have the binding sites of miR-29b-2 (Supplementary Figure 3). To know whether miR-29b regulates the expression of the predicted targeted cytokines, activated CAFs and Hs578Bst fibroblasts (Hs578BstCM) were transfected with miR-29b-1 or miR-29b-2 and the mRNA levels of endogenous cytokines were measured by real time RT-PCR. The results showed that CCL11 and CCL18 mRNA decreased when CAFs and Hs578BstCM were transfected with miR-29b-1, and the mRNA levels of CXCL9, CXCL14 and CXCL17 were slightly decreased (Figure 3A). CCL11 and CXCL14 mRNA decreased when CAFs or Hs578BstCM were transfected with miR-29b-2, but mRNAs of CCL4L1 and CCL4L2 were not changed significantly (Figure 3B).

Next, to confirm that CCL11 and CCL18 were the target genes of miR-29b-1 and CCL11 and CXCL14 were the target genes of miR-29b-2, plasmids with 3′ UTR of CCL11, CCL18 and CXCL14 were constructed. CAFs and Hs578BstCM were transfected with CCL11, CCL18 or CXCL14 3′ UTRs and miR-29b, and luciferase activities were assayed. The results showed that the luciferase activities of wide types of CCL11 and CCL18 3′ UTR in CAFs or Hs578BstCM with miR-29b-1 were much lower than miRNA controls (Figure 3C–3D). The luciferase activities of wide types of CCL11 and CXCL14 3′ UTR in CAFs or Hs578BstCM with miR-29b-2 were much lower than miRNA controls (Figure 3E–3F). CCL11, CCL18 protein levels decreased significantly when CAFs or Hs578BstCM were transfected with miR-29b-1(Figure 3G–3H). CCL11, CCL14 protein levels decreased significantly when CAFs or Hs578BstCM were transfected with miR-29b-2 (Figure 3G–3H). CCL11 and CXCL14 protein decreased in CAFs or Hs578BstCM with miR-29b-2 transfection by western blotting (Figure 3I). When miR-29b-1 or miR-29b-2 was suppressed in MCF-7 cells, CCL11 and CXCL14 were inhibited at mRNA and protein levels (Supplementary Figure 4). So, the data clearly indicated that CCL11 and CXCL14 were the direct target genes of miR-29b in CAFs or Hs579BstCM.

### MiR-29b restoration in CAFs inhibits breast cancer cellular viability and metastasis by targeting CCL11 and CXCL14

**Figure 4:**
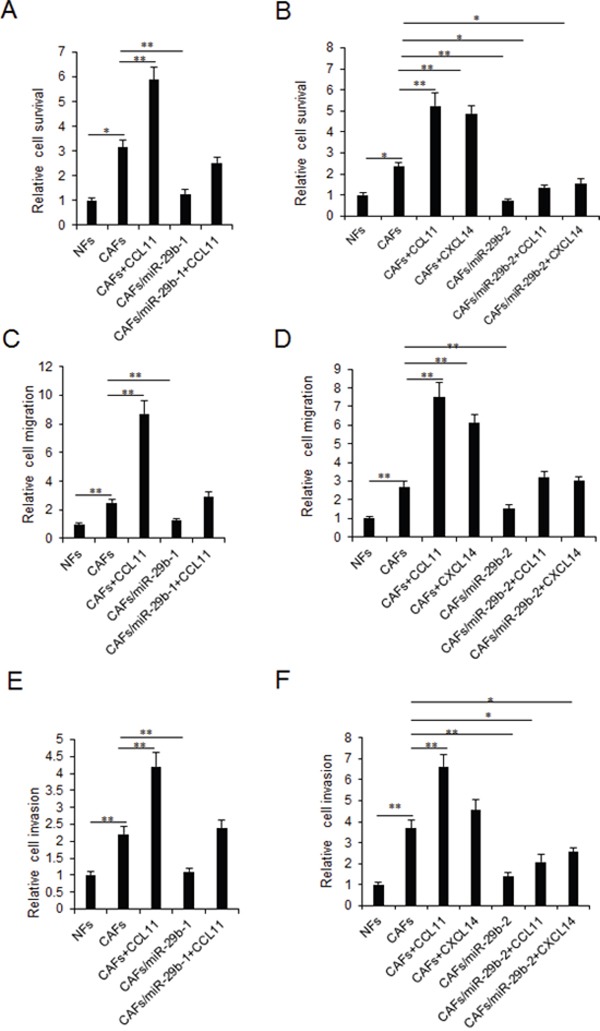
MiR-29b restoration in CAFs inhibits breast cancer cellular viability and metastasis by targeting CCL11 and CXCL14 CAFs were transfected with miR-29b mimics or treated with CCL11 or CXCL14 for 24h and then co-cultured with breast cancer cells. **(A-B)** MTT assay was used to test cell proliferation at day 3. **(C-D)** Cell migration was assayed by transwell chamber system. **(E-F)** Cell invasion was assayed by transwell chamber system. The data were shown as means±s.d. collected from three independent experiments. *: *p*<0.05, **: *p*<0.01.

To further investigate the role of miR-29b in fibroblasts on breast cancer cells, CAFs cells were transfected with miR-29b and then co-cultured with breast cancer cells exposed to CCL11 and CXCL14 (10ng/ml) for 24h. Cell proliferation was examined by MTT assay. It was found that miR-29b could inhibit breast cancer cell survival and also suppressed cell growth by CCL11 and CXCL14 stimulation (Figure 4A–4B). MiR-29b could inhibit breast cancer cell migration via CCL11 or CXCL14 from CAFs (Figure 4C–4D). Breast cancer cell invasion ability was also inhibited by miR-29b, which could suppress CCL11 and CXCL14 enhanced invasion (Figure 4E–4F). When the cells were transfected miR-29-1 or miR-29-2 inhibitors, cell survival ability and migration were enhanced (Supplementary Figure 5).

### CAFs with low level of miR-29b increases breast cancer cell drug resistance by targeting CCL11 and CXCL14

**Figure 5:**
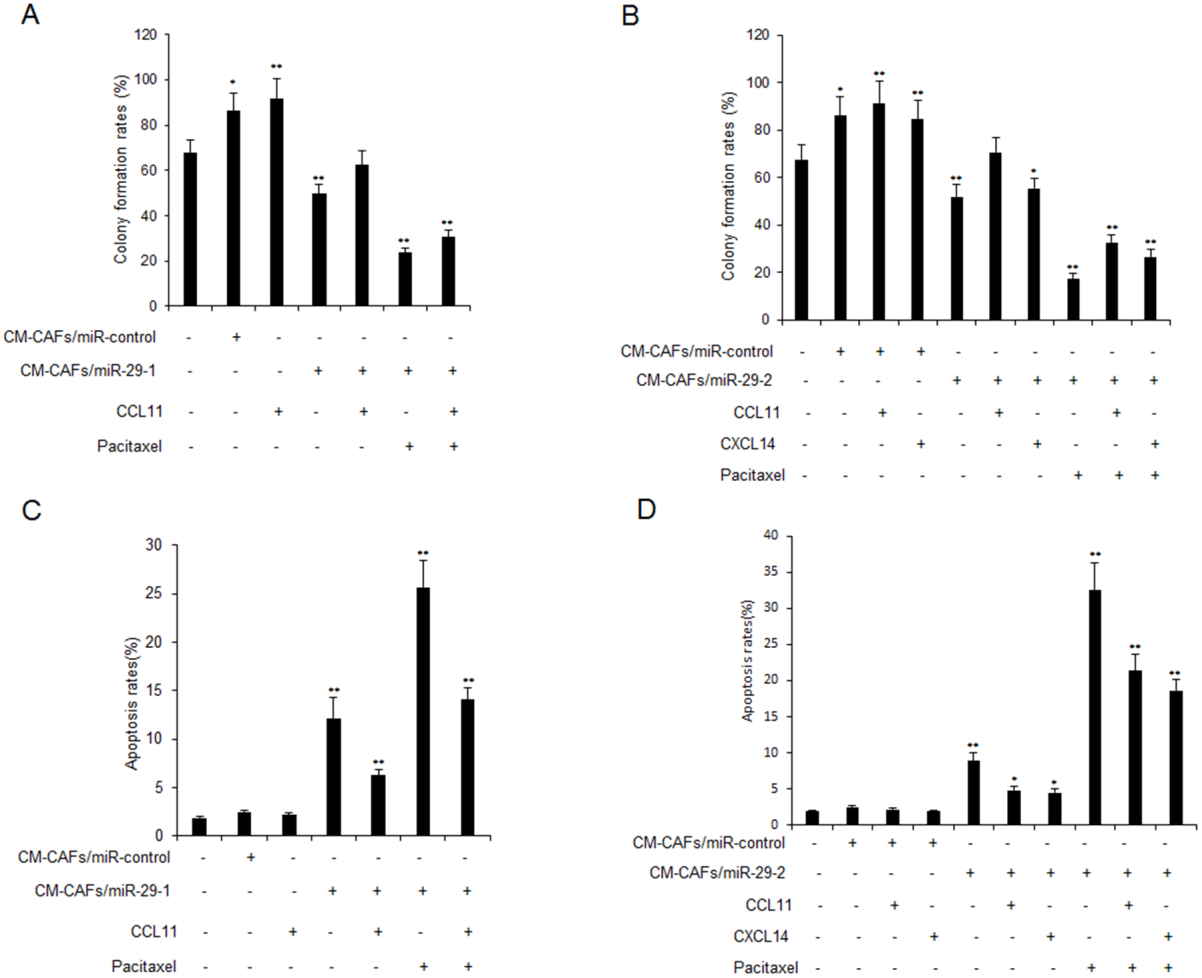
CAFs with miR-29b down-regulation regulates breast cancer cell drug resistance by targeting CCL11 and CXCL14 **(A-B)** MCF-7 cells were treated with CM-CAFs, CM-CAFs/miR-29b-1 or CM-CAFs/miR-29b-2 and then exposed to paclitaxel (10uM) or CCL11 or CXCL14 for 24 hours. Cells were seeded in 6-well plates for two weeks and cell survival ability was evaluated using colony formation assay. **(C-D)** MCF-7 cells were treated with CM-CAFs, CM-CAFs/miR-29b-1 or CM-CAFs/miR-29b-2 and then exposed to paclitaxel (10uM) or CCL11 or CXCL14 for 24 hours. Cells were labeled with AnnexinV and apoptosis was assayed by flow cytometry. The data were shown as means±s.d. collected from three independent experiments. *: *p*<0.05, **: *p*<0.01.

CAFs could induce drug resistance of cancer cells. Here, to know whether there are differences of miR-29b down-regulation on drug resistance of breast cancer cells, MCF-7 cells were treated with CM-CAFs, CM-CAFs/miR-29b-1 or CM-CAFs/miR-29b-2 and exposed to paclitaxel (10uM) or CCL11 or CXCL14. The cell survival rates were examined using colony formation assay. The results indicated that CM-CAFs/miR-29b-1 or CM-CAFs/miR-29b-2 could lead to breast cancer cells growing more slowly than breast cancer cells with CM-CAFs treatment, which had higher survival ability than the parent cells (Figure 5A–5B). Cell apoptosis was also examined in the MCF-7 cells with CM-CAFs, CM-CAFs/miR-29b-1 or CM-CAFs/miR-29b-2 with paclitaxel or CCL11 or CXCL14 treatment for 24 hours. It was shown that MCF-7 cells with CM-CAFs/miR-29b-1 or CM-CAFs/miR-29b-2 showed more apoptosis rates than the cells with CM-CAFs treatment (Figure 5C–5D).

### Lack of miR-29b levels in CAFs activates p38-STAT1 pathway in breast cancer cells

**Figure 6:**
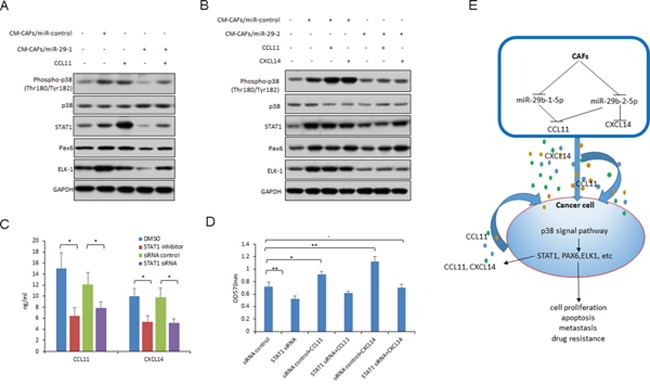
MiR-29b down-regulation in CAFs activates p38-STAT1 in breast cancer cells **(A)** p38 and p38 down-stream gene expression in MCF-7 cells with CM-CAFs or CM-CAFs/miR-29b-1 and CCL11 treatment. **(B)** p38 and p38 down-stream gene expression in SKBR3 cells with CM-CAFs or CM-CAFs/miR-29b-2 and CCL11 or CXCL14 treatment. **(C)** CCL11 and CXCL14 expression in MCF-7 cells transfected with STAT1 siRNA or treated with STAT1 inhibitor. **(D)** Cell proliferation of MCF-7 cells was assayed by MTT method. Cells were transfected with STAT1 siRNA or treated with STAT1 inhibitor and then exposed to CCL11 or CXCL14 for 24h. **(E)** Summary of the study. The data are shown as means±s.d. collected from three independent experiments. *: *p*<0.05, **: *p*<0.01, and -: no significance.

To explore the signal pathway involved in miR-29b stimulation, MCF-7 and SKBR3 cells were exposed to CM-CAFs or CM-CAFs/miR-29b and the protein extraction was for western blotting. We found that p38 was activated in MCF-7 cells with CM-CAFs treatment, however, in the CM-CAFs/miR-29b or STAT1 is one of down-stream transcriptional factor of p38 and it was also inhibited in the breast cancer cells with CM-CAFs/miR-29b or CM-CAFs/miR-29b treatment. CM-CAFs/miR-29b treated cells decreased the activated p38 (Figure 6A and 6B). Other down-stream gene such as ELK1 and PAX6 were also changed.

To know whether STAT1 involves in the chemokine expression of breast cancer cells, MCF-7 cells were transfected with STAT1 siRNA or treated with STAT1 inhibitor, CCL11 and CXCL14 were assayed by ELISA. The results showed that inhibition of STAT1 could reduce the levels of CCL11 and CXCL14 (Figure 6C). When the cells were transfected with STAT1 siRNA and treated with CCL11 or CXCL14, cell proliferation enhancement by CCL11 or CXCL14 was inhibited in the cells with STAT1 down-regulation (Figure 6D). The results from MTT assay indicated that inhibition of STAT1 suppressed the cell survival ability significantly in MCF-7 cells.

## DISCUSSION

Although the stromal cells in the tumor environment including fibroblasts are not malignant, their altered gene expression profiles influence the cellular function and signal pathways of stroma cells, tumor cell behaviors by the communication between tumor cells and stroma. Fibroblasts are the most common cell type found in the tumor microenvironment and usually activated to cancer-associated fibroblasts (CAFs) due to the signals from the microenvironment, which are responsible for the synthesis of proteins involved in the remodeling of the extracellular matrix (ECM) as well as secretion of growth factors that regulate tumor cell proliferation, survival and dissemination. In the present study, we identified that miR-29b was the important molecule for the regulation of chemokines' expression in CAFs.

MiRNAs have been implicated in the activation of fibroblasts. There are reports showed that miR-31, miR-148a and others are down-regulated in activated fibroblasts [9–16]. Previous study showed that miR-29b is down-regulated in prostate cancer and breast cancer [20–21]. Our study showed that miR-29b was down-regulated in CAFs, when miR-29b was introduced into CAFs, the characteristics of CAFs were like NFs (not shown). MiR-29b acts as a tumor suppressor in most cancer types [23–29]. Our results supported that miR-29b plays a suppressor role in tumor stroma.

There are reports shown that fibroblasts involve in the cross-talk of cancer cells and fibroblasts by secreting CCL2 [30–32], CCL5 [33], CXCL1 [34], CXCL6 [35], CXCL12 [36], IL6 [37], CXCL16 [38] and others [23–31]. In the study, we identified that CCL11 and CXCL14 were the target genes of miR-29b in fibroblasts. When miR-29b expression is downregulated in CAFs or activated fibroblasts, CCL11 and CXCL14 expression were significantly higher than the normal fibroblasts. CCL11 and CXCL14 could secret to the outside of the cells and they mainly exist in the extracellular microenvironment. MiR-29b was shown as a negative regulator of CCL11 and CXCL14, but not other chemokines predicted listing in Figure 2. When the CAFs or activated fibroblasts were transfected with miR-29b mimics, CCL11 and CXCL14 expression in the culture media were suppressed. CCL11 and CXCL14 from the CAFs will exert on breast cancer cell and then promotes cell proliferation and metastasis. Further molecular mechanism exploration indicated CCL11 and CXCL14 could active p38-STAT1 pathway in breast cancer cells. Our findings showed that lack of miR-29b expression in CAFs could promote CCL11 and CXCL14 expression, which activate p38 and then promote cell proliferation and metastasis.

**Table 1:** The selected significant miRNAs from the CAFs and NFs

CAFs/NFs>2 meant that miRNAs were significantly up-regulated in CAFs. CAFs/NFs<0.5 meant that miRNAs were significantly down-regulated in CAFs.

In a conclusion, as shown in Figure 6E, our study elucidated that lack of miR-29b expression in CAFs could promote cellular viability and metastasis by activating p38-STAT1 in breast cancer cells by up-regulation of CCL11 and CXCL14 expression in CAFs. STAT1 could promote CCL11, CXCL14 and CCL2 production and then accelerate cell proliferation. This is a positive feedback process. There need more studies to evaluate the function of other miRNAs aberrantly regulated in CAFs and their network. Revealing the molecular mechanisms involved in CAFs-mediated cancer growth and progression may be beneficial for finding new cancer therapy targets.

## MATERIALS AND METHODS

### Cell lines and culture

The breast cancer cell lines were originally purchased from American Type Culture Collection (ATCC, Manassas, VA, USA) and were maintained in Dulbecco's Modified Eagle's Medium containing 10% fetal bovine serum, 100 units/mL penicillin, and 100μg/mL streptomycin. Fibroblasts Hs578Bst were obtained from ATCC and maintained in Hybri-Care Medium (ATCC, Manassas, VA, USA) with 30 ng/ml EGF, 100 units/mL penicillin, and 100μg/mL streptomycin. All the cell lines were cultured in a humidified atmosphere of 95% air and 5% CO_2_ at 37°C.

### Isolation and culture of cancer-associated fibroblasts

Carcinoma associated fibroblasts were isolated referred to the previously reports from five breast cancer tissues [22], which were diagnosed with mammary ductal carcinoma. The experiment was obtained the agreement from the Institutional Review Board of our hospital. The fresh breast cancer tissue samples were washed and kept in PBS containing antibiotics (100 U/ml penicillin, 100 μg/ml streptomycin, 1.5g/ml Fungizone) (Invitrogen Corporation, Carlsbad, CA) at 4°C. The tissues were cutted into about 2 mm fragments and then digested overnight at 37°C with 0.1% collagenase III (Worthington Biochemical Corp., Lakewood, NJ) in DMEM and 10% FBS. The following day, the epithelial cells were separated by centrifuge. The stromal cells were washed in PBS and cultured. All experiments were performed before the 10th passage. In addition, normal fibroblasts (2cm from breast cancer tissues) were isolated according to the CAFs isolation.

### Co-culturing of breast cancer cells and fibroblasts and conditioned medium preparation

CAFs and NFs were co-cultured with breast cancer cells with the ratio at 1:1. Cells were cultured in DMEM/F12 media with 10% FBS supplemented with 10% FBS in a 37°C humidified incubator with an atmosphere of 5% CO_2_ and 95% air for 24 hours, and then washed for three times with PBS and finally cultured in 3ml serum free DMEM/F12 media for 2 hours. Conditioned medium was collected and filtered through a 0.22-μm filter (Merck Millipore, Massachusetts, USA) to remove cellular debris.

### Reagents

Antibodies against MCT-1, MCT-4, FSP1, CAV-1, α-SMA, phospho-p38 (Thr180/Tyr182), p38, PAX6, STAT1, and total β-actin were obtained from Cell Signaling Technology. All other chemicals were purchased from Sigma-Aldrich. Recombinant human chemokines kits were purchased from R&D (Minneapolis, MN, USA).

### Real time RT-PCR

Total RNA was extracted from the cells with the indicated treatment using Trizol reagent (Invitrogen) according to the manufacturer's protocol. RNA was qualified and performed for real time RT-PCR analysis. The primers sequences used in this study were provided in the Supplementary Materials (Supplementary Table 1). The relative miRNA or mRNA levels were calculated by comparing Ct values of the samples with those of the reference, all data normalized to the internal control GAPDH or U6 snRNA.

### Western blot analysis

Cells were lysed in a lysis buffer containing 50 mmol/L TRIS-HCl, pH 7.4, 150 mmol/L NaCl, 0.5% NP40, 50 mmol/L NaF, 1 mmol/L Na_3_VO_4_, 1 mmol/L phenylmethylsulfonyl fluoride, 25 μg/mL leupeptin, and 25 μg/mL aprotinin and clarified by centrifugation (14,000 g for 30 min at 4°C). The protein concentration was determined using the Bradford Coomassie blue method (Pierce Chemical Corp.). Whole-cell lysates were separated by sodium dodecyl sulfate (SDS)-PAGE, transferred onto nitrocellulose, and probed with various primary antibodies and horseradish peroxidase–labeled secondary antibodies. The signals were visualized with an enhanced chemiluminescence detection kit (Promega).

### ShRNA lentivirus vector construction

ShRNA lentiviral particle delivery system was used to generate STAT1 siRNA, and STAT1-silenced tumor cell lines were done according to the manufacturer's instructions (Sigma-Aldrich, Saint Louis, MO, USA). The lentiviral particles were purchased from Sigma (Sigma-Aldrich, Saint Louis, MO, USA). After selection under puromycin (1μg/ml), the knocking down effect in the drug resistant cells was evaluated by western blot.

### Cell proliferation assay

Cells were cultured in 24-well plates with low-glucose (1g/L), low-serum (0.5% FBS) medium (0.5 mL/well) at 37°C. Following the indicated treatments, 10 mg/mL methylthiazolyldiphenyl-tetrazolium bromide (MTT) was added (50 μL/well), and the cells were incubated for an additional 2 hours. The cells were then lysed with a lysis buffer (500μL/well) containing 20% sodium dodecyl sulfate (SDS) in dimethyl formamide/H_2_O (1:1, v/v; pH 4.7) at 37°C for at least 6 hours. The relative number of surviving cells in each group was determined by measuring the optical density (OD) of the cell lysates at an absorbance wavelength of 570 nm.

### Cell colony formation

The cells were harvested, sparsely plated, and were cultured under the normal condition. The medium underwent the replacement at three-day intervals. And then the cells were fixed in 90% ethanol, stained with crystal violet and colonies consisting of at least 50 cells so they were counted ten days later.

### Cell cycle

In 2ml culture medium 2×10^5^ cells/well (6-well plate) were seeded, and cultured for the indicated time before collection. The cells were stabilized with 75% ethanol for 24 h, and dyed with PI, and analyzed with ModFit of flow cytometry.

### Cell apoptosis

For apoptosis assay, the Annexin V straining was quantified by flow cytometric. The cells were plated in a 6-well plate, transfected with the indicated plasmids or siRNA or IGF2 treatment at 24 h later, the complete growth medium were changed to growth medium without serum. At another 24 h later, the cells were collected, washed in cold PBS twice and resuspended in 1×binding buffer at a concentration of 1×10^6^ cells/ml. After that, the cells in 100 μl solution were transferred to a 5 ml culture tube, with 5 μl Annexin V-FITC and 5 μl PI (BD Biosciences) added, and gently vortexed and incubated for 15 min at RT in the dark. And finally, 400 μl 1× binding buffer was added to each tube to be analyzed by flow cytometry within one hour.

### Lentivirus carrying miRNAs

Vectors carrying miRNAs were constructed using the BLOCK-iT pol II miR RNAi Expression Vector Kit with EmGFP (Invitrogen, Carlsbad, CA, USA). The primary miR-29b-1-5p and miR-29b-2-5p sequences with flanking regions were amplified by PCR and then coloned into pcDNA6.2-GW/EmGFP-miR. Lentivirus for miR-29b-1-5p and miR-29b-2-5p was produced using 293T cells by co-transfection with lipofectamine-2000 (Invitrogen, Carlsbad, CA, USA) according to the protocol according to the instruction. MiR-29b-1-5p and miR-29b-2-5p expression were examined using real time RT-PCR.

### Transfection and dual luciferase assay

Cells were seeded on plates and the transfection was carried out in Opti-MEM medium using lipofectamine-2000 according to the manufacturer's protocol (Invitrogen, Carlsbad, CA, USA). The medium was changed after transfection for 5h, and the cells incubated at 37°C for the indicated time. For reporter assays, cells were transfected with the pGL3 basic vector or the control plasmid with co-transfecting with siRNAs. 48h later, luciferase activity was detected using Dual Luciferase Assay System (Promega, WI, USA) with a Sirius luminometer (Berthold Detection System).

### *In vivo* experiment

5×10^6^ CAFs transfected with miR-29-1, miR-29-2 or miRNA control and cells combining with 5×10^6^ MCF7 or SKBR3 cells were suspended in 100 μL phosphate buffered saline and then injected into the fat pads on 6-week-old female athymic nude mice (Shanghai Laboratory Animal Center, Chinese Academy of Sciences, Shanghai, China). Tumor size was measured once a week, and the tumor growth was analyzed by measuring tumor length (*L*) and width (*W*) and calculated with the formula π*LW*^2^/6. All the animal work was conducted in concordance with the guidelines of the Animal Care Committee.

### Statistics

Data were analyzed by SPSS 13.0 software and presented as mean ±SE of at least three independent experiments. Two-tailed Student's t test was used for comparisons of two independent groups. *p*<0.05 was considered statistically significant.

## SUPPLEMENTARY FIGURES AND TABLES


