# Supplementary material for: Down-regulation of miR-29b in carcinoma associated fibroblasts promotes cell growth and metastasis of breast cancer
Source: Oncotarget. 2017 Apr 16;8(24):39559–70. doi: 10.18632/oncotarget.17136 (PMC5503632; doi:10.18632/oncotarget.17136)
Supplement: Supplementary file 1 [file oncotarget-08-39559-s001.pdf]

# Down-regulation of miR-29b in carcinoma associated fibroblasts promotes cell growth and metastasis of breast cancer

## SUPPLEMENTARY FIGURES AND TABLES

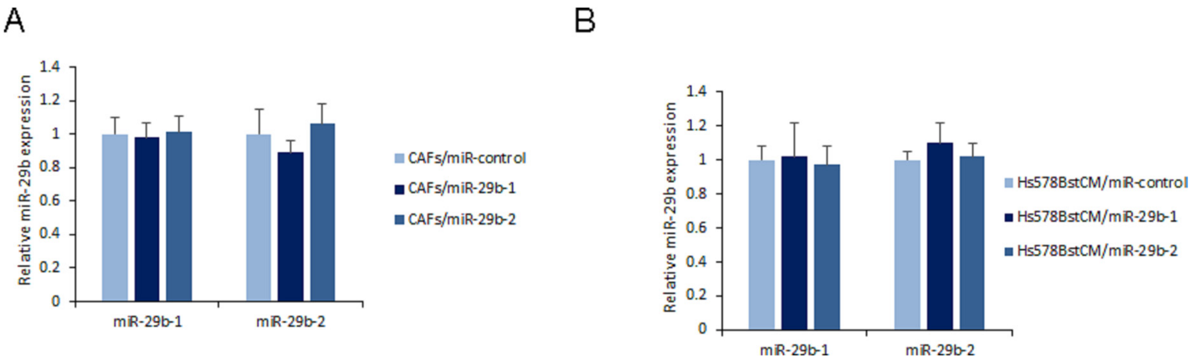


**Supplementary Figure 1: miR-29b expression in breast cancer cells.** The conditioned medium from CAFs/miR-control, CAFs/miR-29b-1, CAFs/miR-29b-2, Hs578BsTCM, Hs578BsTCM/miR-29b-1, and Hs578BsTCM /miR-29b-2 was collected to treat MCF-7 cells. miR-29-1 and miR-29-2 were examined using real time RT-PCR.

<table>
<tr><th></th><th>Predicted consequential pairing of target region (top) and miRNA (bottom)</th><th>Site type</th><th>Context++ score</th><th>Context++ score percentile</th><th>Weighted context++ score</th><th>Conserved branch length</th><th>$P_{CT}$</th></tr>
<tr><td>Position 22-29 of CCL11 3' UTR<br>hsa-miR-29b-1-5p</td><td>5' ...ACCAUUUUUGAAACCAAACCAGA...<br>|||||||<br>3' AGAUUUGGUGGUAUACUUUGGUCG</td><td>8mer</td><td>-0.45</td><td>99</td><td>-0.45</td><td>0</td><td>N/A</td></tr>
<tr><td>Position 420-426 of CCL18 3' UTR<br>hsa-miR-29b-1-5p</td><td>5' ...UAUUAUAUAAAAGGUAAACCAGC...<br>|||||||<br>3' AGAUUUGGUGGUAUACUUUGGUCG</td><td>7mer-m8</td><td>-0.14</td><td>90</td><td>-0.14</td><td>0</td><td>N/A</td></tr>
<tr><td>Position 384-390 of CXCL17 3' UTR<br>hsa-miR-29b-1-5p</td><td>5' ...GGUCUGCCAAUUCACAACCAGAU...<br>||| ||||||<br>3' AGAUUUGGUGGUAUACU-UUGGUCG</td><td>7mer-A1</td><td>-0.13</td><td>89</td><td>-0.08</td><td>0</td><td>N/A</td></tr>
<tr><td>Position 561-567 of CXCL17 3' UTR<br>hsa-miR-29b-1-5p</td><td>5' ...AAAAGCAUACCAAUG--AACCAGAC...<br>|||| ||||||<br>3' AGAUUUGGUGGUAUACUUUGGUCG</td><td>7mer-A1</td><td>-0.19</td><td>94</td><td>-0.12</td><td>0</td><td>N/A</td></tr>
<tr><td>Position 960-966 of CXCL9 3' UTR<br>hsa-miR-29b-1-5p</td><td>5' ...UUAUCCUAUCUCUCCAACCAGAU...<br>||||||<br>3' AGAUUUGGUGGUAUACUUUGGUCG</td><td>7mer-A1</td><td>-0.03</td><td>30</td><td>-0.01</td><td>0</td><td>N/A</td></tr>
<tr><td>Position 1088-1094 of CXCL9 3' UTR<br>hsa-miR-29b-1-5p</td><td>5' ...UAUGGGCAGGAUGGCAACCAGAC...<br>||||||<br>3' AGAUUUGGUGGUAUACUUUGGUCG</td><td>7mer-A1</td><td>-0.03</td><td>30</td><td>-0.01</td><td>0</td><td>N/A</td></tr>
<tr><td>Position 29-35 of CXCL14 3' UTR<br>hsa-miR-29b-1-5p</td><td>5' ...AGAAGGGAAAACUCCAAACCAGU...<br>|||||||<br>3' AGAUUUGGUGGUAUACUUUGGUCG</td><td>7mer-m8</td><td>-0.09</td><td>83</td><td>-0.09</td><td>0</td><td>N/A</td></tr>
</table>

**Supplementary Figure 2: miR-29-1 was predicted to bind the 3'UTR of CCL11, CCL18, CXCL17 and CXCL14.**

<table>
<tr><th></th><th>Predicted consequential pairing of target region (top) and miRNA (bottom)</th><th>Site type</th><th>Context++ score</th><th>Context++ score percentile</th><th>Weighted context++ score</th><th>Conserved branch length</th><th>$P_{CT}$</th></tr>
<tr><td>Position 16-23 of CCL11 3' UTR<br>hsa-miR-29b-2-5p</td><td>5' ...AUAAUCACCAUUUUUGAAACCAA...<br>|||||| |||||||<br>3' GAUUCGGUGGUACA--CUUUGGUC</td><td>8mer</td><td>-0.67</td><td>99</td><td>-0.67</td><td>0</td><td>N/A</td></tr>
<tr><td>Position 54-60 of CCL4L2 3' UTR<br>hsa-miR-29b-2-5p</td><td>5' ...UGUUCUACAGAUUCCAAACCAAA...<br>||||||<br>3' GAUUCGGUGGUACACUUUGGUC</td><td>7mer-A1</td><td>-0.10</td><td>85</td><td>-0.10</td><td>0</td><td>N/A</td></tr>
<tr><td>Position 593-599 of CCL4L1 3' UTR<br>hsa-miR-29b-2-5p</td><td>5' ...UGUUCUACGGAUUCCAAACCAAA...<br>||||||<br>3' GAUUCGGUGGUACACUUUGGUC</td><td>7mer-A1</td><td>-0.04</td><td>69</td><td>-0.04</td><td>0</td><td>N/A</td></tr>
<tr><td>Position 1096-1102 of CXCL14 3' UTR<br>hsa-miR-29b-2-5p</td><td>5' ...GCUAUGUACAUGUCAGAAACCAU...<br>||||| |||||||<br>3' GAUUCGGUGGUACA--CUUUGGUC</td><td>7mer-m8</td><td>-0.29</td><td>98</td><td>-0.29</td><td>0</td><td>N/A</td></tr>
</table>

**Supplementary Figure 3: miR-29-2 was predicted to bind the 3'UTR of CCL11, CC4L2, CC4L1 and CXCL14.**

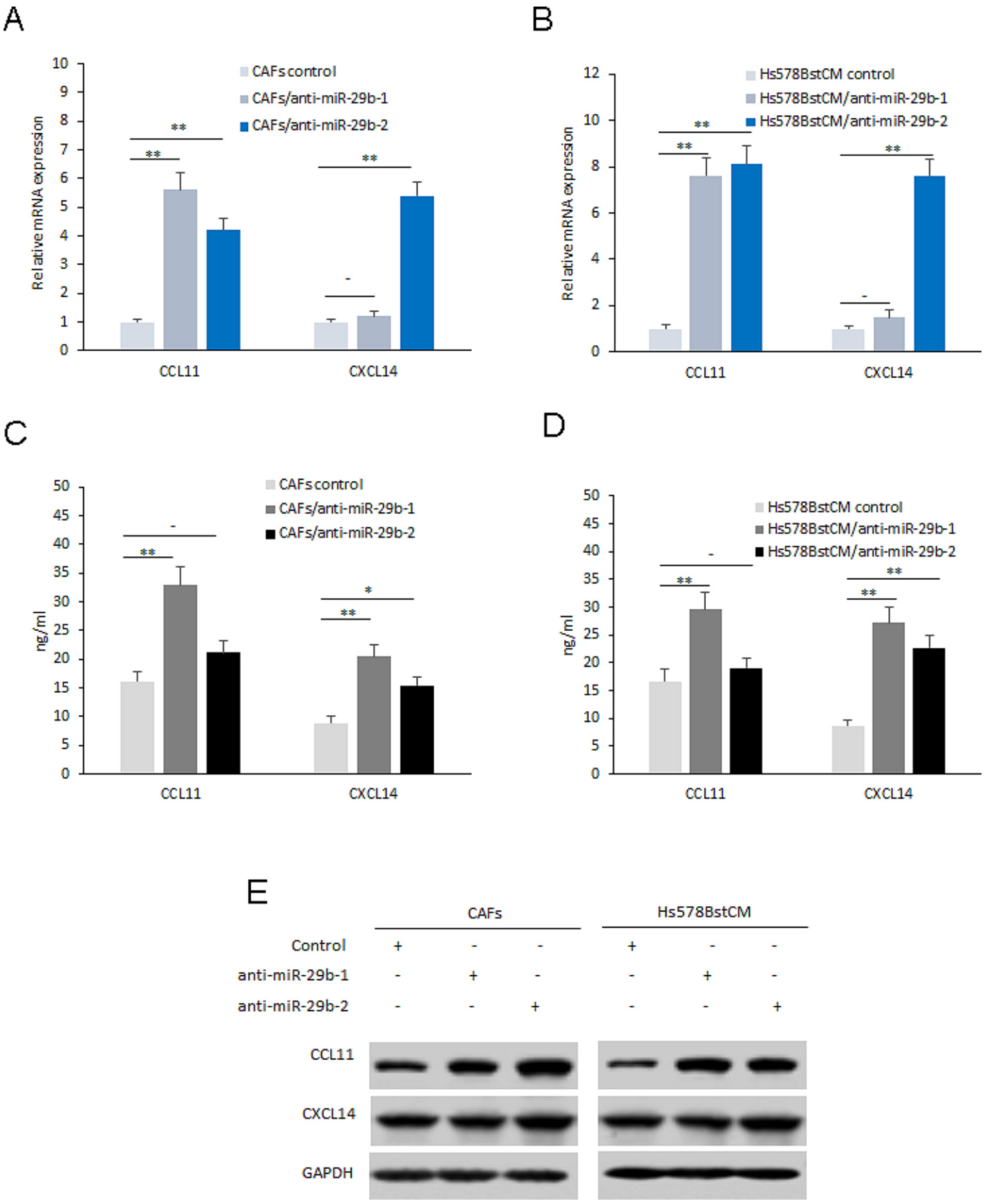


**Supplementary Figure 4: (A)** Inhibition of miR-29b increased CCL11 and CXCL14 mRNA in CAFs. Cells were transfected with miR-29b inhibitor or the control for 48 hours, and collected for real-time PCR. **(B)** Inhibition of miR-29b increased CCL11 and CXCL14 mRNA in Hs578BsT cells activated by the cancer cell conditioned medium (Hs578BsTCM). Cells were transfected with miR-29b inhibitor or the control for 48 hours, and collected for real-time PCR. **(C-D)** Cells were transfected with miR-29b-1, miR-29b-2 inhibitors for 48 hours, then collected for ELISA. **(E)** Cells were transfected with miR-29b-1, miR-29b-2 inhibitors for 48 hours, then collected for western blotting. The data presented are shown as means±s.d. collected from three independent experiments. *: $p<0.05$, **: $p<0.01$, and -: no significance.

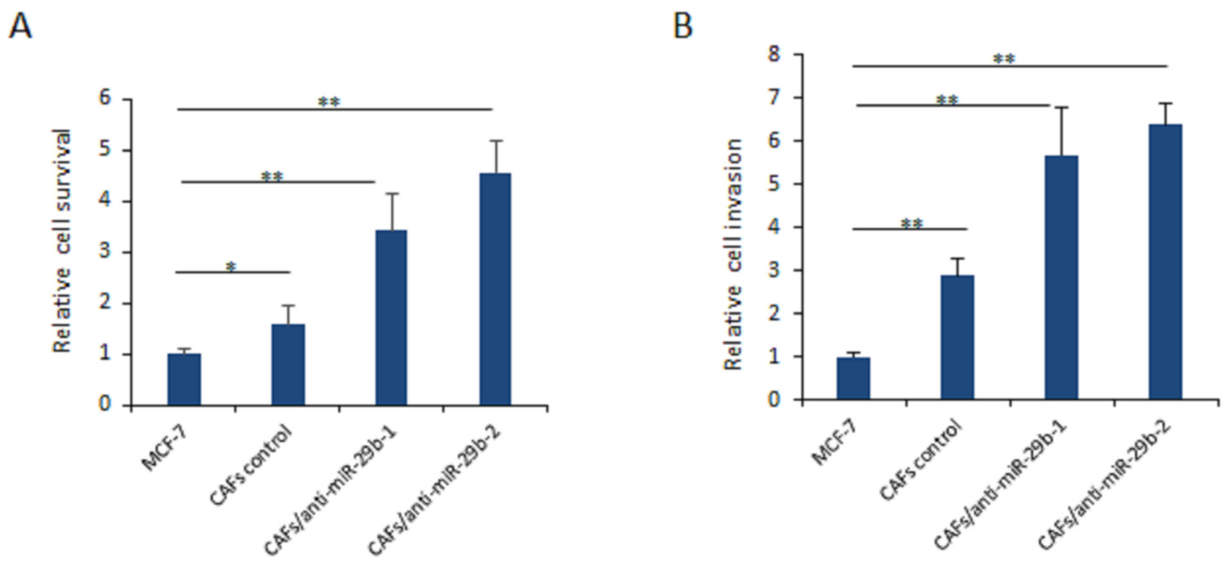


**Supplementary Figure 5:** Inhibition ofmiR-29b promoted MCF-7 cell survival **(A)** and invasion **(B).** *: $p$<0.05, **: $p$<0.01.

**Supplementary Table 1: miRNA array from the CAFs and NFs**

| Up-regulated miRNAs | CAFs/NFs | Down-regulated miRNAs | CAFs/NFs |
|---|---|---|---|
| has-miR-211-3p | 4.73 | MiR-760 | 0.32 |
| has-miR-452-5p | 3.82 | has-miR-237 | 0.19 |
| has-miR-33a-3p | 5.67 | has-miR-204-5p | 0.34 |
| has-miR-182 | 2.45 | has-miR-210 | 0.23 |
| has-miR-22 | 3.12 | has-miR-1233 | 0.28 |
| has-miR-223-3p | 3.76 | has-miR-1-3p | 0.46 |
| has-miR-199a-5p | 2.86 | has-miR-499 | 0.18 |
| has-miR-155 | 2.19 | has-miR-26b | 0.42 |
| has-miR-320 | 2.05 | has-miR-101 | 0.37 |
| has-miR-351 | 2.12 | has-miR-125a | 0.31 |
| has-miR-92 | 2.43 | has-miR-652-3p | 0.24 |
| has-miR-545-5p | 2.09 | has-miR-148a | 0.27 |
| has-miR-106b | 2.20 | has-miR-9-3p | 0.21 |
| has-miR-143 | 2.67 | has-miR-223-3p | 0.38 |
| has-miR-1248 | 4.78 | has-miR-29b | 0.12 ## |
| has-miR-4344 | 2.46 | has-miR-582-5p | 0.29 |
| has-miR-145 | 2.27 | has-miR-200b | 0.16 |
| has-miR-941 | 2.75 | has-miR-146a | 0.23 |
| has-miR-374a-5p | 2.14 | has-miR-301a | 0.40 |
| has-miR-142-5p | 2.91 | has-miR-2278 | 0.28 |
| has-miR-1912 | 3.36 | has-miR-519d | 0.17 |
| has-miR-19a | 3.18 | has-miR-631 | 0.32 |
| has-miR-345 | 7.89 | has-miR-1204 | 0.44 |
| has-miR-4478 | 2.82 | has-miR-3673 | 0.30 |
| has-miR-7565 | 5.45 | has-miR-890 | 0.23 |
| has-miR-205 | 7.12 | has-miR-2467-5p | 0.25 |
| has-miR-17 | 6.41 | has-miR-569 | 0.21 |
| has-miR-6077 | 3.92 | has-miR-3661 | 0.35 |
| has-miR-937-3p | 3.70 | has-miR-7-3p | 0.26 |
| has-miR-20a | 6.08 | has-miR-188-5p | 0.18 |
| has-miR-328 | 5.21 | has-miR-96-5p | 0.45 |
| has-miR-324-3p | 4.11 | has-miR-548q | 0.25 |
| has-miR-4785 | 7.13 | has-miR-4309 | 0.34 |
| has-miR-548-3c | 9.24 | has-miR-181b | 0.46 |
| has-miR-655-5p | 5.51 | has-miR-10b | 0.38 |

**Supplementary Table 2: Primers for real time RT-PCR**

| Gene | Primers (5'-3') |
|---|---|
| miR-29b-1 | Forward: 5'-ACACTCCAGCTGGGGCTGGTTTCATATGGTGG-3'<br>Reverse: 5'-CTCAACTGGTGTCGTGGA GTCGGCAATTCAGTTGAG |
| miR-29b-2 | Forward: 5'-ACACTCCAGCTGGGCTGGTTTCACATGGTG-3'<br>Reverse: 5'-CTCAACTGGTGTCGTGGA GTCGGCAATTCAGTTGAG-3' |
| U6 | Forward: 5'-CTCGCTTCGGCAGCACA-3'<br>Reverse: 5'-AACGCTTCACGAATTTGCGT-3' |
| CCL11 | Forward: 5'-GCAGCTGCCTTCAGCCCC-3'<br>Reverse: 5'-ACCAGGTCTATGAAGTACC-3' |
| CCL18 | Forward: 5'-TGCACAAGTTGGTACCAA-3'<br>Reverse: 5'-CCAGTCGTGTCTAGACGG-3' |
| CXCL14 | Forward: 5'-GCCCCTCCGGTCAGCATG-3'<br>Reverse: 5'-AGGACTGGAGCCATGGAC-3' |
| CXCL17 | Forward: 5'-GCTGATGTCCATGGTCTCTAG-3'<br>Reverse: 5'-TAACGACCGTCCGAGACCTTA-3' |
| CCL4L1 | Forward: 5'-CTAGTAGCTGCCTTCTGCTC-3'<br>Reverse: 5'-CAAGTCAAGGTCCAGTATGTGCA-3' |
| CCL4L2 | Forward: 5'-CGTGACTGTCCTGTCTCTCCTCG-3'<br>Reverse: 5'-GTCCAGTATGTGCATGAGGAC-3' |
| CXCL9 | Forward: 5'-AGTGCAAGGAACCCCCAGTA-3'<br>Reverse: 5'-TAAAAGCTTGAAAGTCTTGA-3' |
| GAPDH | Forward: 5'-ACCACAGTCCATGCCATCAC-3'<br>Reverse: 5'-CCACCACCCTGTTGCTGTAG-3' |
